# Socio-Psychological Impacts of Hydraulic Fracturing on Community Health and Well-Being

**DOI:** 10.3390/ijerph17041186

**Published:** 2020-02-13

**Authors:** Mehmet Soyer, Kylen Kaminski, Sebahattin Ziyanak

**Affiliations:** 1 Department of Sociology, Social Work & Anthropology, Utah State University, 0730 Old Main Hill, Logan, UT 84322-0730, USA; kylen.kaminski@aggiemail.usu.edu; 2 Department of Social Science, University of Texas-Permian Basin, 4901 East University, Odessa, TX 79762, USA; ziyanak_s@utpb.edu

**Keywords:** hydraulic fracturing, socio-psychological impacts, health, well-being, fracking, community cohesion, community change, othering, us vs. them

## Abstract

At the core of the hydraulic fracturing (fracking) debate is the level of perceived risk involved with extractive industries, such as the release of toxic and carcinogenic chemicals, increased population growth, and truck traffic. However, industry supporters of fracking acclaim the benefits of oil and gas drilling, such as energy independence and economic gains. In this study, we examine the perceived impacts of hydraulic fracturing (fracking) on community health and well-being based on interviews with anti-fracking activists in Denton, Texas who were active in the “anti-fracking” community organization, Frack Free Denton (FFD). Emergent from the interviews, we discuss the socio-psychological stressors these community members experienced following the introduction of hydraulic fracturing in the region. Some of the major socio-psychological impacts included perceived physical health risks through anxiety surrounding toxins and carcinogens that may be released through this process. Participants also discussed stress put on community relations, primarily through the form of an “us vs. them” mentality related to the support for, or opposition to, fracking in the community. Moreover, we found anxiety and stress surrounding trust in community members’ relationships with governing bodies, such as the federal government, state government, and local governments. This research will allow for a more comprehensive understanding of how fracking can impact the socio-psychological well-being of the community.

## 1. Introduction

In this study, we examine that hydraulic fracturing can impact the socio-psychological well-being of residents. Even though this research is still in its early stages, researchers are starting to note a relationship between fracking taking place in communities and its socio-psychological effects on residents [1]. Possible factors contributing to socio-psychological alterations are linked to loss of land, chemical use, changing dynamics of the community, population increase, and uncertainty of future outcomes. The overall socio-psychological health of community members can have significant implications on the productivity of the community, along with the overall social and individual health of community members, which suggests a need for socio-psychological health research related to communities experiencing hydraulic fracturing. For the purposes of this research, we define “socio-psychological health” as one’s well-being pertaining to dimensions of both their mental (including emotional) and social health. This type of health is influenced by the environmental changes introduced by fracking through various stressors, impacting both personal and social health in this community. This is evidenced by the issues that community members face and have to deal with when challenges to social cohesion and socio-psychological health in a community occur when looking at fracking-related studies.

Though not specifically related to fracking, Albrecht et al. identify the concept of distress resulting from environmental change around one’s homeland, which they termed “solastalgia” [2]. The research focuses on the correlation of the psychological well-being, specifically distress and anxiety, and the destruction or possible negative impact to a piece of land. Following data analysis, results indicate increased levels of ecosystem distress syndromes and increases in similar human distress syndromes in areas that have experienced significant environmental changes where individuals feel attached to their land [2].

Some preliminary research has also identified socio-psychological impacts resulting from hydraulic fracturing [1]. Current literature on the subject has led researchers to find that fracking is “spanning neurological, psychopathological, interpersonal, and existential concerns,” and “there appears to be an array of levels of socio-psychological functioning that are deleteriously affected by the fracking process and industries and their aftermath” [1]. For example, 59 different health impacts and 13 stressors are linked with hydraulic fracturing [3]. There is no complete argument regarding the perceived impacts of hydraulic fracturing (fracking) on community health and well-being.

Other stressors have been found in similar studies. For example, in Australia, Morgan et al. (2016) found that community members who are exposed to hydraulic fracturing, “…exhibited clinically significant levels of psychological morbidity” [4]. The results from this study indicate that there are two unique stressors present in the population residing around these sites which contribute to higher levels of anxiety and depression [4]. These stressors include concerns about the impacts that oil extraction might have on land operations and privacy and the second pertaining to the possible health impacts on the environment and local community [4].

Economic issues and Denton residents’ rights surrounding the topic of fracking can also be stressors for some residents living within close proximity of these sites. In a study of Denton, Texas, researchers found that much of the economic gains, 68% of the mineral rights, belonged to people outside of Denton resulting from the fracking process not staying in the city of Denton, leaving residents even more worried about the true benefits of fracking in their community [5].

One activist, Tammy, mentioned “These mineral owners from out of town. They don’t see the daily life. They don’t breathe the air, drink the water. They don’t have the capacity to care the way that we do in town. So, we have to start prioritizing surface property owners if we’re going to protect our land, protect our world and have any kind of sustainable future.”

In parallel with these studies, Denton residents took action to resolve their social problems bordered by fracking in their residential areas with social activism [6]. Finally, during fracking, 2 to 10 million gallons of water are mixed with sand and 80 to 300 tons of harmful and harmless substances. Their results showed that more than half of these compounds could potentially distress the brain and nervous system [7]. 

With the introduction of a controversial oil extraction method, it would not be unfounded to assume that social stressors will become present within the community, of which will likely have both negative and positive impacts on social cohesion. This could be exhibited through various ways but likely in situations where there are disagreements between community members. The introduction of environmental issues, or even perceived environmental issues, can also lead to issues between community members, as well as instigating personal stresses to the individuals that perceive the changes. There have also been findings that suggest that with fracking often comes feelings of powerlessness, intimidation, exploitation, and a general feeling of corruption in those who are community leaders [8]. Having hydraulic fracturing present in a community has an effect on community members’ perceived quality of life, which is a result of the stress and extreme emotional pressure that fracking can induce [8]. Moreover, the power struggle between the pro-fracking group and anti-fracking group over hydraulic fracturing resulted in a polarized community in Denton, TX. Researchers found out that “their [Frack Free Denton and Denton Taxpayers for Strong Economy] characterizations of both the in-group and the other further highlight the polarizing nature of hydraulic fracturing debates in local contexts, providing insight into the way local debates shape community practices” [9].

## 2. Methods

We transcribed all recorded in-depth, semi-structured interviews, and we coded the data for common themes and attitudes toward our observed eight main themes and six subthemes. Our findings and qualitative inquiry are presented as a medium for the voices of participants to be heard, which is valuable in the qualitative approach [10].

The researchers used purposive sampling to recruit potential participants from the audience at the town hall meeting on drilling ordinance. Purposive sampling is followed by the snowball sampling method. At this point, the researchers asked the participants to share the researchers’ information with their social networks to engage with many other possible participants.

We addressed the following questions during the interviews in order to reach our research objectives, such as “What do you think of fracking in Denton?”, “When and how did you hear about fracking?”, “Please briefly summarize what it is that you know about fracking?”, “Please talk to me about any associated problems about fracking?”, “Please tell me about possibility of safe fracking in Denton?”, and “Please tell me about the possibility of fracking anywhere safely?”.

There were eight themes that consisted of general stress and anxiety, health, isolation, optimism, pessimism, helplessness, social health, and social frustration. We also generated six subthemes: environmental heath, physical health, social cohesion, social fracturing, us vs. them, and social activism.

The group that we studied was located in a small city in northeastern Texas close to Dallas, called Denton, Texas. At the time the interviews were conducted in 2015, the impact of fracking was relatively new to the community, and Denton community did not see emerging social problems as their concern. Therefore, the community was not at the awareness stage.

The protocol of this study was approved by the Institutional Review Board (IRB) at Texas Woman’s University to ensure the following of the IRB standards and ethics. Each participant was provided a consent form and was also verbally informed of any risks associated with participation in this research. The participants’ confidentiality and anonymity were maintained by giving a pseudonym name for each participant. Our participants voluntarily took part in this study. Since the participants for this study were from the anti-fracking group (Frack Free Denton), we didn’t experience any recruitment issues in this study.

Researchers utilized transcripts from in-depth, semi-structured interviews to code for common frames surrounding the stressors that may be affecting the community socio-psychological health. The interviews were conducted with a local grassroot anti-fracking group named Frack Free Denton (FFD). There were a total of 15 interviews conducted with this group. In terms of participants’ characteristics, of these interviews, 7 participants were female and the remaining 8 were male. 

The transcriptions were then coded using NVIVO 11 software. During the coding process, researchers looked for characteristic phrases related to emerging socio-psychological dimensions, such as “I was anxious about …” or “I don’t know what I was feeling but I didn’t want to deal with ….” Dimensions included: general stress and anxiety, environmental and physical health, isolation, optimism, pessimism and helplessness, social cohesion, social fracturing, us vs. them, and social activism. Each statement was sorted into the developing dimensions (anxiety, physical health, social activism, etc.), sometimes being sorted into multiple constructs depending on their multiple applicability. 

## 3. Results

### 3.1. General Stress and Anxiety

Members in this community felt increased amounts of stress and anxiety as compared to periods of time prior to the introduction of fracking. This was evidenced in approximately 69 statements made by FFD members. These statements included: 

Clair stated that “They started drilling and fracking out there on our property and we couldn’t stop them from doing that because we didn’t own the minerals.” Another community member, Diego, asserted that “So, there was noise, and there was truck traffic, and there was fumes and there was lights and that when it became abundantly clear that even after two sets of revisions to our Gas Well Ordinances, that they were still inadequate.”

### 3.2. Health

#### 3.2.1. Environmental Health

The environmental health of the community was often questioned by community members and was also a common source of stress and/or anxiety. This was manifested in statements surrounding the purity of water in the region, the increased rates of respiratory disorders and other physical defects, and the death of animals due to drinking from tainted wells. 

Liam: “Probably the biggest news that came out of that story was how much more wastewater was coming up with unconventional drilling.”

Sarah: “Fracking itself uses up way too much water.”

The odors and fumes that were produced by fracking sites also provided not only environmental concerns but would also be a likely stressor in conjunction with the increased vehicle traffic and light pollution. Across all transcripts, there were 40 statements in regard to the environmental health impact of fracking.

#### 3.2.2. Physical (Personal) Health

Physical health issues and the increased prevalence of multiple ailments and physical disorders (such as asthma) were also the source of stress and anxiety for a number of members of the community.

Sarah: “I’ve had impacts, health impacts when I’m around [fracking].”

Throughout reading the transcripts, there were also several mentions of the immobilization of medical personnel and their weariness to get involved, which increased the lack of trust that community members had with the medical community. There were also instances of statements made by community members that had correlated the rate of increased physical ailments with the individual’s proximity to fracking sites.

Clair: “I know when they were doing wells over by my house. There were older people who lived on that street who were having more incidents of respiratory difficulties or having to use their emergency medications whenever they were fracking.”

### 3.3. Isolation

One of the consequences that the introduction of fracking on this community has had, which is often also a result of the othering that occurred in this community, was the isolation that people felt. It was interesting, however, that the isolation that occurred was far less prevalent than was anticipated (only two statements across all interviews). This could likely be due to social cohesion that occurred within the FFD group and amongst anti-fracking supporters. 

Abby: “So I think that was alienating for us right and that gave us a feeling of dispossession or underrepresentation.”

### 3.4. Optimism

Interestingly, one of the findings of this study was that optimism occurred with community members slightly more than pessimism. This falls in line with the finding that there were few statements of, and feelings surrounding, isolation. Tammy explains how her message motivates her and her social movement members’ prospects for their cause. “We got a message just all the time on Facebook from all over the world. People saying, ’Good job. You gave us hope.’ Wherever, Eastern Europe or wherever all these places.”

The optimism found in this study often stemmed from the feeling that there was community support for members of FFD, even if it was only from other members. The in-group connection that many members felt provided positive feelings surrounding this movement’s progression. 

Holly: “I think [local public officials have] been receptive. I mean, my feeling was ‘they know this is not good for the community’.”

### 3.5. Pessimism

While pessimism did occur throughout the community members’ transcripts, it was far less frequent than statements surrounding optimism (13 statements across only seven interviews). For example, Clair states that “We had lost the battle, they got to put the wells in. We did everything we could during that process of fracking to protect ourselves and we could not. And there was just a hopeless feeling in our neighborhood and people just disconnected.”

Much of the pessimism that occurred was in regard to the receptiveness of land owners, as well as public officials. There were commonly feelings that officials were not making decisions to benefit the community as a whole, or its ecosystem, but to rather further their own personal economic standings. Community members also felt that there was little support from state officials and the need for the community to ban the fracking process in the community. 

Clair: “It’s not even really about the ban anymore. I don’t expect to win. We’ll have to give up some time because we can’t just keep being sued so many times. The city can’t afford it. We’re not gonna win, this is Texas.”

### 3.6. Helplessness

Frack Free Denton members also somewhat frequently reported feelings of helplessness in regard to reaching out for public official support regarding the introduction of hydraulic fracturing in their community (40 instances in the transcripts). This was important to look at, as feeling helpless in a large community situation where you have group support could further fracture the community and have mental health consequences (as well as physical health consequences). Some members are repetitively articulate in the need of such support. They also complain about the public being so quiet regarding the depths of sharing their thoughts associated with hydraulic fracking.

Randy: “There was just a deafening silence about it [fracking] and that both intrigue me and upset me because it seems to me, they’re not doing their duty as guardians of the health of public. Nobody says a thing. Nobody does anything at the level. Why you are at the doctors, mobilize on this. Now, understand that in earlier years, a group of doctors did sign a petition. But then like quit. There was no collective activity on the part of the health professionals.”

The feelings of helplessness could easily manifest themselves into symptoms of anxiety and stress that cause the aforementioned issues with community members. Therefore, feelings of helplessness experienced by community members can cause overlap between different constructs looked at in this study and demonstrate comorbidity between the constructs.

Clair: “We called the State several times to come and do air monitoring but they would never show up… Because a lot of that land is owned by the ranchers who own the minerals – so even when I would ask doctors out there to get involved, to try and help, they would say they couldn’t because their patients were mineral owners and they could possibly lose their patients.”

### 3.7. Social Health

Once community members have established othering, social health issues such as marginalization, hatred, social exclusion, social distance from new identity seeking and development, and stigmatization will affect each person’s psychological health and social purpose in the community, key principles of social harmony. Othering will be the main instigator of hatred and the production of labeling [11]. Kristen emphasizes on the eco-terrorism by addressing othering and social distance in her explanation. She states “I’ve been even called an “eco-terrorist” so many times. There was someone in Mansfield who said that he needed a bodyguard because the eco-terrorists were going to get him.”

#### Social Cohesion

While social cohesion in the community as a whole was decreased throughout the introduction of fracking to the community and the different social responses to it, we can see that there were specific areas in which social cohesion increased. Community members would come together based on their ideas surrounding fracking, which brought some community members further together and increased the positive aspects of these social interactions.

Kelly: “I think in my own neighborhood it created a sense of solidarity because almost all of us on that little cul-de-sac had signs of Frack Free Denton. And so, you know, it’s exciting when you’re all in it together. Right. You’re all fighting the man together and do things.” 

Tammy: “It’s more like one-on-one neighborly stuff. We all go to city council meetings and we speak publicly there.”

### 3.8. Social Frustration

#### 3.8.1. Social Fracturing

The social fracturing that occurred in Denton mainly occurred between the pro-fracking community members and anti-fracking community members. As a result of this social fracturing, we saw that the rate of which people othered community members (distancing themselves socially from them) increased. 

Matt: “Most of my friends are not members of chambers. In fact, I don’t think anybody down here is a member of chambers. They are the fools, but there could be a lot of foolish people.”

#### 3.8.2. Us vs. Them

This construct was by far the most prevalent in the study. Us vs. Them, or “othering”, as it pertains to this research, is the act of social distancing or demonizing of an opposition group (or outside group). In this study, we looked at the interactions between the anti-fracking group “Frack Free Denton” (FFD) and their distancing from any other outside group. Across all of the interview transcripts, in total, we saw 98 instances of this taking place. This was examined through statements such as:

George: “We live in a plutocracy and they are the owners, nothing exclusively but the five or six major fossil fuel industries that are currently terrorizing the planet. They more or less own America, this is their political mechanism.”

Randy: “The industry isn’t even really contributing to our town.”

In statements such as this, we can see that the FFD members are trying to distance their group from that of the oppositional group. This further creates cohesion within the group and further pushes the idea, within the group, that the fracking industry (we also see this in regard to politicians, fracking supporters, and other anti-fracking groups) is in opposition to their values. 

This process is also done to the political members of the community, with FFD members often holding the idea that they (the politicians) are working with the oil industries to further their agenda or are not assisting locals that are against fracking being introduced to the community in any way. 

One interesting finding to come out of the othering analysis has to do with the amount of othering that FFD members often engaged in when discussing other anti-fracking groups and organizations. This phenomenon was much more common than anticipated, as members of the FFD group would often “other” from these other supportive organizations, making statements in regard to the thought that they were not there to assist the FFD movement but rather to further their own organizations and ideas. This type of othering would also occur if FFD members believed that the ways of other anti-fracking groups were too extreme or were associated with politically undesired (in the community) organizations. 

#### 3.8.3. Social Activism

Unsurprisingly, social activism was quite common across all transcripts. The process of making “calls to arms” was quite prevalent across community members in their recollection of the anti-fracking movement they were involved in. It also pertained to the amount of activist activities that members of the FFD group engaged in during this anti-fracking movement. 

Mark: “My degree of activism, activism in the sense there’re different levels of it. I will take you back to when, like my car, that was a level of activism, that’s way for people to… My idea was there if you’re stuck in traffic and you see me, you have something to think about. If it’s a bumper sticker on sometimes you won’t read it but if you see 400 hundred bumper stickers, you’ll pay attention.”

Randy: “But we did everything and I think, we were probably all agreed that the thing that had the most impact was going door-to-door. We knocked on lots and lots of doors.”

## 4. Discussion

According to the information that was gathered, there were various effects that the introduction of fracking had on the community. Most emboldened was the presence of community fracturing through the process of “othering” (us vs. them) that took place in this group. All of the individuals that were interviewed made various statements to distance themselves not only from the opposing groups (those who supported fracking) but also many of the groups that were present to support them. There were ideas that the groups that were there to help had their own agendas and were not actually there to support their group/organization. The members of this group also distanced themselves, socially, from community officials, especially those above the municipal level (state officials, etc.). Unsurprisingly, there was a large amount of othering taking place against the fracking industry. 

Another factor that caused great amounts of stress and anxiety in this community had to do with general health (both to the community members and the environment surrounding them). Some of the individuals interviewed had backgrounds in health fields, all of which voiced concerns about health in regard to their community members. There were also stories surrounding the environment, in which whole fields of cows were killed due to, what they believed, was contaminated water from the fracking sites. Our findings contribute to previous research findings with reference to increasing mistrust and community stress. The crucial point is that local residents are standing for their own rights against the fracking corporations through grassroot activism [12]. Our findings confirm that lack of governmental ruling and implementation linked to fracking resulted in rising community concerns, such as mistrust and community stress [13,14].

Many of the community members also voiced a lack of control in their community. They explained that many of the fracking sites were extremely close to their homes, or schools, which many of the local officials endorsed (in their perceptions). In this parallel, our findings are in consensus with previous researches’ findings in terms of an increasing loss of control of local residents’ private and public lives. For example, Perry (2012) [15], Poole and Hudgins (2013) [16], and Willow (2014) [14] state that demoralized and devastated persons who reported negative experiences with fracking were more likely to lose their control over their lives and possessions. On the other side, those experiences were the foremost reasons for their communal awareness.

## 5. Conclusions

The purpose of this research was to examine Denton local community awareness and have a greater understanding of the impacts that can be caused by the introduction of fracking. This study contributed to pertinent literature by discussing the effects of hydraulic fracking from psychological and sociological standpoints. Moreover, this qualitative study delivered a deep and contextualized understanding of the socio-psychological features of the local residents’ experiences and involvement through the hydraulic fracturing (fracking) debate in Denton, TX. In this study, there were a number of limitations. We need to note that we might have a possible selection bias through purposive and snowball sampling. One of the potential limitations of this study is that we could not generalize the findings beyond Denton.

This research has further highlighted the socio-psychological impacts that hydraulic fracturing (fracking) has had on a local Texas community and its residents. Our identification of the socio-psychological impacts in this community supports prior research on this topic, as well as adds new issues of concern. Our results indicate that socio-psychological impacts caused by othering and helplessness are directly related to the local residents’ social well-being. 

One of our contributions to the existent literature is to recognize the socio-psychological impacts of “othering” (us vs. them) that occur when the fracking process is introduced to the community of Denton. While this dynamic created further cohesion within the “us” part of the groups, overall, it fractured the community.
